# Economic evaluation in chronic pain: a systematic review and de novo flexible economic model

**DOI:** 10.1007/s10198-015-0720-y

**Published:** 2015-09-16

**Authors:** W. Sullivan, M. Hirst, S. Beard, D. Gladwell, F. Fagnani, J. López Bastida, C. Phillips, W. C. N. Dunlop

**Affiliations:** BresMed Health Solutions, Sheffield, UK; CEMKA-EVAL, Bourg-la-Reine, France; University Castilla-La Mancha, Madrid, Spain; Swansea University, Swansea, Wales, UK; MundiPharma International, 194 Cambridge Science Park, Milton Road, Cambridge, Cambridgeshire CB4 0AB UK

**Keywords:** Economic evaluation, Chronic pain, Transparency, Modelling assumptions, I10

## Abstract

There is unmet need in patients suffering from chronic pain, yet innovation may be impeded by the difficulty of justifying economic value in a field beset by data limitations and methodological variability. A systematic review was conducted to identify and summarise the key areas of variability and limitations in modelling approaches in the economic evaluation of treatments for chronic pain. The results of the literature review were then used to support the development of a fully flexible open-source economic model structure, designed to test structural and data assumptions and act as a reference for future modelling practice. The key model design themes identified from the systematic review included: time horizon; titration and stabilisation; number of treatment lines; choice/ordering of treatment; and the impact of parameter uncertainty (given reliance on expert opinion). Exploratory analyses using the model to compare a hypothetical novel therapy versus morphine as first-line treatments showed cost-effectiveness results to be sensitive to structural and data assumptions. Assumptions about the treatment pathway and choice of time horizon were key model drivers. Our results suggest structural model design and data assumptions may have driven previous cost-effectiveness results and ultimately decisions based on economic value. We therefore conclude that it is vital that future economic models in chronic pain are designed to be fully transparent and hope our open-source code is useful in order to aspire to a common approach to modelling pain that includes robust sensitivity analyses to test structural and parameter uncertainty.

**Electronic supplementary material:**

The online version of this article (doi:10.1007/s10198-015-0720-y) contains supplementary material, which is available to authorized users.

## Introduction

Chronic pain has been defined as persistent pain, lasting at least 3 months [1], and can present in the course of many diseases, including cancer, trauma, neuropathy and osteoarthritis [2]. The pharmaceutical treatment of chronic pain is highly dependent on the needs of the individual patient, the severity and frequency of their pain, and also on local clinical practices. The overall approach to the treatment and management of chronic pain is best considered with reference to the World Health Organisation’s (WHO’s) ‘three-step’ ladder. The three-step ladder outlines the movement of patients with uncontrolled chronic pain through various treatment stages, based on the use of: standard oral analgesia (e.g. aspirin, paracetamol) or anti-inflammatory drugs (e.g. non-steroidal anti-inflammatory drugs); milder opioids (e.g. codeine, tramadol); and finally the more potent stronger opioids (e.g. morphine, oxycodone, fentanyl) [3]. However, patients who suffer from chronic pain continue to have unmet need [4]. In addition, the treatment paradigm for the control of moderate to severe chronic pain may be shifting towards a new wave of non-opioid drug treatments that will sit outside of the standard use of current opioid treatment options. As international treatment standards for effective pain control continue to develop, it will become increasingly important to be able to appraise the cost-effectiveness of competing pain therapies (both new and old) in a consistent, transparent and robust way to understand the true value of these treatments.

While the attributes and management of specific diseases associated with chronic pain, such as fibromyalgia and osteoarthritis, and different analgesic requirements for neuropathic versus other chronic pain may have justifiably led to modelling heterogeneity, clinical differences between the management of (1) malignant and (2) non-malignant chronic pain have generally not warranted different approaches to economic evaluation. This study uses the term chronic pain to describe pain of malignant or non-malignant causation.^1^ Economic evaluations of pharmaceutical treatments in chronic pain have used modelling approaches to justify price premiums for novel products, but have faced a number of key hurdles and challenges in how best to describe the overall treatment pathway for patients. First, the nature of the individual drug treatments can lead to difficulties in the management of adverse event profiles, including but certainly not limited to the impact from chronic constipation and severe nausea [5–7]. Side-effect differences between therapies are not necessarily powered for in clinical studies, making meaningful comparison difficult. Second, the efficacy of treatment is extremely individualised and as such is difficult to assess and categorise across patients. A significant proportion of patients will be expected to achieve at best only a partial control of their pain symptoms, even if treatments are well tolerated. This level of expected variability in both the adverse effect (AE) profile and pain response means that treatment discontinuation, patient monitoring and treatment switching (both across and within drug class) are all key aspects of the ‘real-life’ clinical management of chronic pain [8, 9]. These aspects need to be recognised and fully considered when conducting evaluations of economic value. Third, the designs of clinical trials are rarely based on multiple lines of therapy and are also seen to apply strict protocol-driven levels of adherence, which can ultimately underestimate the scale of ‘real-life’ treatment discontinuation and switching. Finally, the equivalence design of many clinical trials, with effectively unlimited dose titration, means that detecting any meaningful difference in pain control (where patients have remained on therapy) is extremely difficult to achieve.

All these factors may have led to variability and limitations in the approaches and modelling structures used in the economic evaluation of pharmaceutical treatments in chronic pain. This may have restricted access to treatments to patients in great need and in the longer term may discourage innovation in novel analgesic therapies. The UK National Institute for Health and Care Excellence (NICE) has guidelines in place for opioid use in palliative care [10], and the Guideline Development Group (GDG) were critical of the potentially serious limitations of the previous models they identified [6, 11, 12]. The challenges to economic evaluation in this area that have led to variation in modelling approaches may have also severely limited the usefulness of findings from previous studies.

In this study we first conducted a systematic review of the published economic literature to help identify, summarise and explore the key areas of variability and limitations in the modelling approaches used in economic evaluations of treatments for chronic pain. The results of the literature review were then used to support the development of a de novo economic model structure. A full set of scenario and sensitivity analyses were then conducted in order to highlight and explore how such a model structure can be best used to address the key areas identified in the systematic review, where the care pathway may need to consider treatment withdrawal and switching and where parameter uncertainty remains because of a lack of robust data.

The de novo model structure can be potentially used as a ‘reference case’ for future economic models for pain therapy and to guide future practice. To help with accessibility and applicability to different country settings, an effort has been made to make the model fully flexible and transparent, with the open-source code (in the programming language R) being provided as supplementary material. This is intended to allow other researchers to easily adapt and apply the model to further progress the development of health economic models in pain therapy.

## Methods and materials

### Systematic review

The systematic literature review was designed to identify peer-reviewed English language economic evaluations of oral, nasal or transdermal pharmacological treatments for chronic pain, published since January 2000. The search strategy was developed through a scoping exercise and targeted the databases MEDLINE, EMBASE, Health Technology Assessment, NHS Economic Evaluation Database and EconLit. The scope of the review was economic evaluations for which the pathway of chronic pain treatment and not the nuances of a particular disease drove the modelling approach. For this reason, though the search strategy was broad, studies specific only to osteoarthritis, low back pain, neuropathic pain, fibromyalgia and post-surgery pain were excluded from the final review. All searches were performed within the University of Sheffield’s School of Health and Related Research (ScHARR) during April 2014. Full details of the search strategy are available as supplementary material.

#### Identified economic evaluations and model designs

The search identified 12 published relevant economic evaluation studies [5–7, 10–18]. From these, eight original model structures were identified [5–7, 10, 13, 15, 17, 18], comprising one individual-level discrete event simulation (DES) model [12] and seven cohort-level state-transition (Markov) models. The most common country settings were the UK [10, 13, 17] and Germany [11, 12, 15]; other studies have been set elsewhere in Europe or the USA. Studies identified in the review focussed on opioid-based treatments, including regimens of morphine, oxycodone, tapentadol, tramadol, oxycodone, fentanyl and buprenorphine, sometimes in combination with naloxone. Perhaps due to non-inferiority trial designs for opioid trials, previous models have assumed an equal analgesic effect across comparators and have captured differences in effectiveness in terms of rates of withdrawal and adverse effects only.

The key features of the model designs used are summarised in Table 1.

**Table 1:** Summary of published economic models in chronic pain

References	Country setting	Chronic pain type	Therapies considered	Time horizon	Cycle length	Dose titration included	Treatment lines	Model type	Costs	Assumption of efficacy (analgesic effect)
Coluzzi and Ruggeri [18]	Italy	Non-malignant	Oxycodone/naloxone; tapentadol; oxycodone	1 year	15 weeks	No	1 line	Markov	Direct	Equivalent efficacy
Dunlop et al. [17]	UK	Non-malignant	Oxycodone/naloxone; oxycodone	43 weeks	1 week	No	1 line	Markov	Direct	Equivalent efficacy
Frei et al. [7]	Denmark	Non-malignant	Fentanyl; morphine	1 year	30 days	No	2 lines	Markov	Direct	Equivalent efficacy
Hass et al. [15]	Germany	Non-specific	Buprenorphine; fentanyl; oxycodone; morphine	6 years	1 year	No	1 line	Markov	Direct	Equivalent efficacy
Ikenberg et al. [13]. Structure later used by: Obradovic et al. [14]	UK	Non-malignant	Tapentadol; oxycodone	1 year	4 weeks	Yes	3 lines	Markov	Direct	Equivalent efficacy
Neighbors et al. [6]. Structure later used by: Lehmann et al. [11]; Grenier et al. [12]; Hauber et al. [16]	USA	Non-specific	Fentanyl; morphine; oxycodone	1 year	NA	Yes	2 lines	Decision tree	Direct	Equivalent efficacy
NICE [10]	UK	Malignant	Morphine; oxycodone; fentanyl; buprenorphine	1 year	1 week	No	2 lines	Markov	Direct	Equivalent efficacy
Neil et al. [5]	USA	Non-malignant	Tapentadol; oxycodone	1 year	NA	Yes	2 lines	Discrete event simulation	Direct	Equivalent efficacy

Though 5 of 12 studies reported systematic searches to identify input data [6, 10, 12, 13, 18], in the absence of data on key elements of the care pathway, previous studies have been reliant on expert opinion to inform key parameter and structural modelling decisions. This may have led to variation in structural design across the previous models. Some authors, such as the NICE GDG [10], have favoured less complex model designs, which reflect the availability of robust data, while others have designed their model to be sufficiently complex to capture care pathways.

#### Key design themes

Key model design decisions and variations across models include: (1) the number of treatment lines considered; (2) the choice of treatments across consecutive treatment lines; and (3) the approach used to capture the initial titration and stabilisation phase of opioid treatment. Approaches to these decisions have varied across previous studies, but no study has explored the implications of uncertainty in their choice of modelling approach for study findings. This is a key focus in the development of the reference case de novo economic model structure described in this study.

Expert opinion data are uncertain and analyses using such data to inform cost-effectiveness estimates should incorporate this uncertainty into their sensitivity analysis. While previous models have reported deterministic and probabilistic sensitivity analyses, the scale of uncertainty assumed around expert parameter estimates may not have been sufficiently wide to fully capture uncertainty. Extrapolation of data over time also requires further assumptions, and the consequences from the exact choice of analytical time horizon has not always been robustly tested in previous models.

In addition, future generations of pharmacological therapies for chronic pain may offer analgesic improvement, and previous models may not have been designed to directly capture and reflect the value from such outcomes (again with a focus on non-inferiority in efficacy driven from clinical trial designs).

Derived from the systematic review of economic models, the key areas or model design themes for exploration in this study can be summarised as follows:

1. time horizon;
2. titration and stabilisation;
3. number of treatment lines;
4. choice of treatment across consecutive treatment lines;
5. potential for value from analgesic improvement across interventions;
6. implications of uncertainty around parameter estimates.

### Reference model

#### Model transparency and coding

A flexible decision analytic model was initially programmed in Microsoft Excel^®^ to appraise competing pharmacological treatments for chronic pain. The model was then replicated in the program R [19] to validate the model and to put it into a code-based format that can be easily shared, hence allowing full transparency. To replicate presentation of sensitivity analysis results the R package “ggplot2” was used [20]. The model design was based on findings and key themes identified from the systematic review, and the model was designed explicitly to have the capacity to explore key areas of uncertainty. The authors hope this model, which is referred to from here as the ‘reference model’, will be useful to practitioners as a point of reference in guiding future model design in the area, and the model is made freely available as supplementary material in the form of R code. The model is also presented as supplementary material without the full sensitivity analysis code and in Microsoft Excel so it is readily accessible and understandable to researchers new to the model.

#### Model structure

In line with the majority of previous models [6, 7, 10, 13, 15, 17, 18], the reference model is a state-transition Markov cohort model. The model structure has been designed and built with sufficient complexity and flexibility to consider the range of uncertainty in design features identified through the systematic literature review.

Though the care pathway for chronic pain may be complex and heterogeneous, data limitations may preclude meaningful benefit from discrete event or discrete individual simulation approaches for contemporary chronic pain models. In addition, the use of DES models in HTA assessment is not universally supported by reimbursement bodies and can create additional hurdles in decision making. For this reason, the potential advantages from a DES approach have not been considered within the design of the reference model, primarily in order to retain a widely applicable and adaptable model structure.

**Fig. 1:**
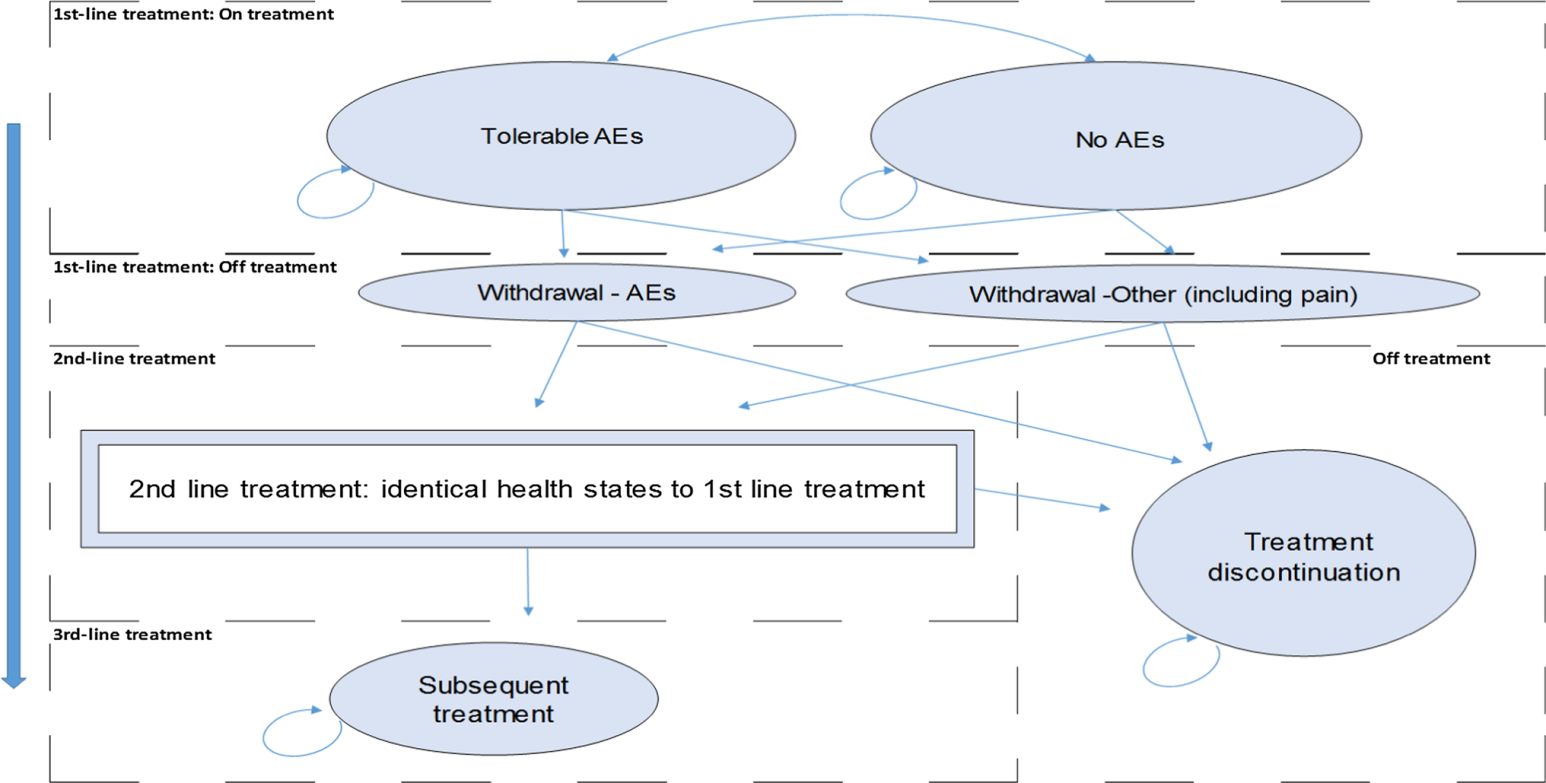
Model structure—one treatment arm

The reference model structure for one treatment arm is illustrated by Fig. 1. Each model state is associated with a cycle cost and utility value, and movements between model states are determined by the model structure and input data. A hypothetical cohort of patients with chronic pain enter the model upon treatment initiation, while on first-line treatment patients are distributed across two model states depending on whether or not they are experiencing tolerable treatment-related AEs.

##### Time horizon

The time horizons adopted by previous models have been similar and limited. All except one study used a time horizon of 1 year or less and assumed zero mortality. The remaining study used a 6-year time horizon, but did not model treatment- or disease-related mortality [15]. The study assessed opioid-related fractures as a key health outcome and included consideration of the higher incidence of death following fractures [15]. Only one model [7] employed trial data with follow-up equal to the model length; the majority of studies relied on assumptions to extrapolate beyond trial endpoints, and the implications of such assumptions for model results were not always robustly tested. In line with previous models and in order to accurately capture the available input data, the reference model uses a cycle length of 1 week and a time horizon of 1 year as the base case.

##### Titration and stabilisation

Some previous studies have modelled the titration and stabilisation phase(s) as a separate model state(s), typically lasting a month [6, 13] or in one case 8 weeks [12]; others have not considered the cost and health-related quality of life (HRQL) implications of the initial stages of therapy [7, 10, 15, 17, 18], perhaps because the implications of the titration and stabilisation phase for model outcomes have been similar across treatment arms when two or more opioids have been compared. The model structure in Fig. 1 does not include a separate state(s) for titration and stabilisation, but the model is designed so that the cycle probabilities of withdrawal and treatment costs can be adjusted for the first 4 weeks of first-line treatment, allowing the importance of titration assumptions to be tested in the structural sensitivity analysis.

##### Withdrawal

Treatment withdrawal is a feature of the typical patient care pathway in chronic pain. Of the eight unique published models structures identified in the literature review, six considered the health economic consequences of treatment withdrawal [5–7, 10, 13, 18]. These models varied however in the scope of reasons for withdrawal and the assumptions on the subsequent management of these patients.

In the reference model, patients may withdraw from first-line treatment because of either: (1) intolerable AEs or (2) other reasons including insufficient pain relief. Following one model cycle in either of the transitory states, labelled “Withdrawal—AEs” and “Withdrawal—Other (including pain)” in Fig. 1, patients either move to second-line treatment or discontinue, in effect leaving the care pathway. The model states for second-line treatment are identical to those for first-line treatment; while on treatment, patients may or may not experience tolerable treatment-related AEs and patients may withdraw from second-line treatment because of intolerable AEs or for other reasons including insufficient pain relief.

##### Treatment lines/subsequent treatment

The majority of previous models (5 of 8) have captured outcomes for multiple treatment lines [5–7, 10, 13], and the consequences of treatment withdrawal and subsequent care are greatly important for health and cost outcomes. The reference model was therefore designed with the capacity to capture outcomes over multiple treatment lines. However, as shown in Table 1, the number of treatment lines considered varied across previous models, and it was felt important to test the effect of different structural assumptions in this area. Following second-line treatment withdrawal, the reference model has the flexibility to either assume all patients discontinue treatment or, as explored in the scenario analysis and depicted in Fig. 1, assume a proportion of patients move into an absorbing state representing subsequent treatment. The states “Subsequent treatment” and “Treatment discontinuation” are the two absorbing states in the model.

### Model inputs

#### Treatment comparison/analysis perspective

In this exploratory analysis, the reference model is used to compare a hypothetical newly developed oral therapy (referred to herein as the novel therapy) to a representative comparator morphine. Morphine is widely used internationally as a reference treatment in the management of chronic pain and recommended as a first-line treatment for pain in palliative care in the UK [10]. The country setting for three studies in the review was the UK [10, 13, 17] and the perspective on costs in this study is that of the UK National Health Service and Personal Social Services (NHS and PSS) (the healthcare payer). Due to fact that there were a number of UK models in the literature, including an HTA, the UK was chosen as the example country for the reference case. It is anticipated that the UK example can be readily applied and adapted to other geographies, particularly similar health systems within Europe. The perspective on health outcomes is that of direct health effects.

In the absence of robust evidence for second-line treatment choice following withdrawal from morphine, the model assumes second-line oral controlled-release oxycodone therapy across treatment arms, though morphine as second-line treatment following novel therapy treatment is also explored as a scenario.

Although morphine is recommended as a first-line treatment for chronic pain in certain settings in the UK [10], the more novel oral controlled-release oxycodone has exhibited a preferable tolerability profile in comparison to morphine in previous studies [12] and is increasingly used internationally in clinical practice—often as first-line therapy [13]. It is likely that a therapy entering the market would exhibit superior efficacy in comparison to the more effective drug, at a price premium. The comparison assumes the hypothetical novel therapy is six times as expensive and yields a 30 % improvement in levels of tolerable AE rates and withdrawal rates versus oxycodone.

Model inputs were sourced from the economic evaluation studies identified in the systematic literature search. The best available data for each model parameter were sought from previous studies. These data provide an initial basis to the de novo model and hypothetical exploratory analysis and are a good indication of the available data in this area. All model input estimates were validated by a UK clinical expert. Due to the short time horizons considered, model health outcomes and costs were not discounted. A half-cycle correction was applied to cost and health outcomes.

#### Adverse event and withdrawal rates

Table 2 describes the cycle probability estimates for tolerable AEs, withdrawal due to intolerable AEs or other reasons, and treatment discontinuation used in the exploratory analysis.

**Table 2:** Parameter estimates for cycle probabilities of tolerable AEs, treatment withdrawal and care discontinuation

Parameter description	Estimate	Source
Cycle probability tolerable AE, morphine	0.436	Calculated from [12]^a^
Cycle probability withdraw because of AE, morphine	0.056	Calculated from [12]^b^
Cycle probability withdraw because of other reason, morphine	0.013	Calculated from [12]^b^
Cycle probability tolerable AE, oxycodone	0.464	Calculated from [13]^c^
Cycle probability withdraw because of AE, oxycodone	0.033	Calculated from [13]^d^
Cycle probability withdraw because of other reason, oxycodone	0.002	Calculated from [13]^d^
Cycle probability tolerable AE, novel therapy	0.324	Assumption: proportional reduction of 0.3 relative to oxycodone
Cycle probability withdraw because of AE, novel therapy	0.023	Assumption: proportional reduction of 0.3 relative to oxycodone
Cycle probability withdraw because of other reason, novel therapy	0.002	Assumption: proportional reduction of 0.3 relative to oxycodone
Cycle probability discontinue after failed 1st-line treatment	0.050	Assumption

^a^Seven-day probability of nausea/vomiting calculated from 28-day rate plus assumed constant risk of constipation

^b^Seven-day probability calculated from midpoint of 28-day probability range

^c^Assumed constant risk of experiencing adverse effects from 105-day rate

^d^Seven-day probability calculated from 105-day probability

The cycle probabilities of tolerable AEs and withdrawal rates attributable to morphine were calculated from data reported by Greiner et al. [12], the most recent data available for these parameters (also later used by Hauber et al. [16]). The tolerable AEs considered in the reference model are nausea/vomiting and chronic constipation. Greiner et al. [12] reported 28-day rates for these AEs and withdrawal rates because of AEs and other reasons (lack of efficacy) separately for morphine patients. Following Greiner et al. [12], a duration of 7 days for nausea/vomiting is assumed. For chronic constipation, the duration of adverse effects was not reported, and it is assumed that the percentage of patients reported to experience mild chronic constipation over 28 days was equivalent to the probability of constipation with morphine in any model cycle. There were no data on the proportion of patients who experienced nausea/vomiting and constipation concurrently; it was assumed that the cycle probabilities of each adverse event are additive, resulting in a cycle probability of experiencing tolerable AEs on morphine of 43.6 %, as shown in Table 2.

The corresponding cycle probabilities for second-line oxycodone were calculated from Ikenberg et al. [13], who reported 105 day rates for (1) tolerable AEs, (2) withdrawal because of AEs and (3) other reasons (lack of efficacy) for a large sample of patients (*n* > 1000) receiving oxycodone as a second-line treatment. Though patient numbers and transitions between health states were clearly reported by Ikenberg et al. [13], details of the duration of adverse effects were not clear. The cycle probability of experiencing tolerable AEs on oxycodone was assumed equivalent to the proportion of oxycodone patients who experienced tolerable AEs over the 15-week trial reported by Ikenberg et al. [13], 46.4 % as shown in Table 2.

There is a risk of patients giving up on the care pathway and discontinuing treatment rather than attempting alternative treatment options after a failed therapy, though data on this risk are lacking in previous models. This exploratory analysis assumed a 5 % cycle probability of treatment discontinuation treatment following first-line treatment failure.

#### Resource use and cost estimates

Table 3 shows the cost data used in the model. All costs have been inflated to 2013 values where appropriate [21]. Drug costs for morphine and oxycodone were taken from the British National Formulary (BNF) 67 [22]. Following NICE guidance [10], a maintenance dose of 60 mg morphine per day was assumed, and the use of Morphgesic^®^ SR, MST Continus^®^, Zomorph^®^ and Filmarine^®^ SR (the four modified-release 12-hourly oral preparations listed in the BNF 67 [22]) was assumed to be evenly distributed. Taking an unweighted average of pack prices for the pill doses available up to 30 mg for each oral preparation, the price of morphine therapy was calculated as £2.63 per week. Following Dunlop et al. [17], a maintenance dose of 32.2 mg oxycodone per day was assumed. The price of a 56-tab pack of generic oxycodone 10 mg is £22.86 and the price per milligram does not vary by pill strength [22]; the price of oxycodone therapy was therefore calculated as £9.20 per week.

**Table 3:** Parameter estimates for model costs

Parameter description	Estimate	Source
Treatment cost per cycle, morphine	£2.63	BNF 67 [22]; NICE [10]
Co-medication cost per cycle, morphine	£2.26	NICE [10]; Curtis et al. [21]
Treatment cost per cycle, oxycodone	£9.20	BNF 67 [22]; Dunlop et al. [17]
Co-medication cost per cycle, oxycodone	£0.04	Dunlop et al. [17]; Curtis et al. [21]
Treatment cost per cycle, novel therapy	£55.21	Assumption: 6 times oxycodone treatment cost
Co-medication cost per cycle, novel therapy	£0.03	Assumption: proportional reduction of 0.3 relative to oxycodone
Adverse event cost per cycle	£6.99	NICE [10]; Curtis et al. [21]
Cost associated with withdrawal	£106.91	NICE [10]; Curtis et al. [21]
Treatment discontinuation cost per cycle	£18.50	Assumption: Half discontinued patients visit GP weekly; Curtis et al. [21]

The weekly costs of concomitant laxatives to prevent constipation while receiving morphine, as well as the cycle cost associated with tolerable AEs, are those reported by NICE [10], the only previous economic evaluation to consider morphine for chronic pain from a UK NHS cost perspective. Weekly concomitant drug costs for oxycodone are those reported by Dunlop et al. [17], a recent UK study that took a micro-costing approach to estimate the treatment and concomitant medication costs associated with oxycodone. The weekly cost of novel therapy treatment is assumed to be six times the cost for oxycodone, while the cost of concomitant laxatives is reduced by 30 % compared to oxycodone. The one-off cost associated with withdrawal is that used by NICE [10], comprising a general practitioner (GP) surgery visit, 10 min with a medical consultant, a 20-min visit from a Community Nurse and a GP telephone consultation. Following treatment discontinuation, 50 % of patients are attributed the cost of a weekly GP visit [21].

#### Utility estimates

The quality and applicability of utility data varied across previous health economic studies. Ikenberg et al. [13] reported EQ-5D utility data from 15-week trials of competing opioid regimen patients (*n* > 1000) with chronic pain [23]; these data have also been used to capture the HRQL in two other studies in the review [14, 18]. Elsewhere, Dunlop et al. [17] reported the only other economic evaluation in which generic preference-based HRQL data from patients in the effectiveness trial were available to inform the analysis. However, the data, from over 300 non-cancer pain patients [24] were analysed by treatment arm and time since randomisation and reported by categories incompatible with the reference model structure [17].

In the model built to inform guidelines for opioid use in palliative care [10], the NICE GDG used an HRQL estimate for controlled pain from a standard gamble study of 95 patients with chronic non-cancer pain [25], a source used to inform utility assumptions in two further economic studies [12, 16]. The earliest economic evaluations in the review [6, 11] derived utility estimates for controlled and uncontrolled pain from another standard gamble study, whose sample of 114 participants was drawn from the general population [26]. More recently, Neil et al. used SF-6D data from a multisite study of 96 non-cancer pain patients to inform utility estimates for adequate and inadequate pain relief [5]. Of the two remaining economic studies in the review, Hass et al. [15] used general population HRQL data to capture baseline utility and assigned fracture-related utility decrements, while Frei et al. [7] did not consider HRQL, measuring outcomes as ‘days of good pain control’.

Table 4 shows the utility data used in the model. The categories reported by Ikenberg et al. [13], derived from EQ-5D utility data from 15-week trials of competing opioid regimens reported by over 1000 chronic pain patients, map to the four model states associated with one treatment line in the reference model and are reasoned to be the most appropriate available from the studies reviewed for this exploratory analysis. These estimates are used for first-line treatment states.

**Table 4:** Parameter estimates for model utility values

Parameter description	Estimate	Source
Utility, on treatment, no AEs	0.695	Ikenberg et al. [13]
Utility, on treatment, tolerable AEs	0.583	Ikenberg et al. [13]
Utility, withdrawn from treatment due to AEs	0.503	Ikenberg et al. [13]
Utility, withdrawn from treatment due to other reasons	0.405	Ikenberg et al. [13]
Utility multiplier, failed 1st-line treatment	0.900	Assumption
Utility multiplier, failed 2nd-line treatment	0.800	Assumption

Patients' HRQLs are likely to fall as they experience failed treatment lines. To reflect this in the absence of a robust estimate of this effect, the model applies a multiplier of 0.9 to the estimates shown in Table 4 for corresponding second-line model states. Treatment discontinuation is attributed the lowest utility estimate in Table 4, adjusted by an assumed multiplier of 0.8 to reflect the negative effect of successive failed treatments upon HRQL. These assumptions are however easily challenged; the importance of assumptions about lasting HRQL effects of failed treatment lines is explored as a scenario, as described in “Scenario analyses”.

### Scenario and sensitivity analyses

#### Scenario analyses

The exploratory analysis using the reference model was based on a primary analysis using a defined base case scenario: scenario 1 in Table 5. An additional six modelling scenarios (scenarios 2–7 in Table 5) were used to test the sensitivity of reference model outcomes to structural and parameter uncertainty not routinely analysed in economic models of chronic pain.

**Table 5:** Reference model—scenario descriptions

Reference	Description
Scenario 1: “Base case”	The model time horizon is 1 year and two lines of treatment are considered
	Morphine is compared to the novel therapy as a first-line treatment
	The cost and HRQL implications of drug titration and stabilisation are not modelled
	After withdrawal from 1st-line therapy, patients on either model arm either discontinue treatment or switch to oxycodone treatment
	After withdrawal from 2nd-line therapy, all patients are assumed to discontinue treatment
Scenario 2: “3rd-line treatment with morphine”	Explores the consequences of different assumptions about subsequent treatment lines for model results, as scenario 1 with the exception that: Following withdrawal from 2nd-line treatment, 90 % of patients move to a subsequent 3rd-line treatment. The cycle costs attributed to the “Subsequent treatment” health state are set to morphine treatment; the utility tariff attributed to this health state is the average of the four utility estimates in Table 4, multiplied by 0.8 to represent an assumed reduction in patient HRQL (having experienced two failed treatment lines)
Scenario 3: “Morphine as 2nd-line treatment on novel therapy arm”	Explores the consequences of different assumptions about treatment pathways following 1st-line treatment across both model arms, as scenario 1 with the exception that: Novel Therapy patients are assumed to switch to morphine as opposed to oxycodone as a 2nd-line therapy
Scenario 4: “Titration and stabilisation”	Explores the consequences of different assumptions about titration and stabilisation, as Scenario 1 with the exception that: For the first 4 weeks of 1st-line treatment, drug doses and AE probabilities are adjusted in line with clinical data from previous studies (Ikenberg et al. [13] reported doses of around two-thirds of the maintenance dose and withdrawal rates over twice as high as those observed during maintenance therapy, during the first 4 weeks of treatment in their study). Withdrawal rates are doubled and treatment costs multiplied by 0.65 in the first four model cycles
Scenario 5: “Improvement in analgesic effect”	Explores the consequences of different assumptions about achieving pain control superiority, as scenario 1 with the exception that: Utility values for patients receiving 1st-line novel therapy are increased by 5 % to reflect improved levels of pain control when on treatment and responding. Future generations of pharmacological therapies for chronic pain may offer analgesic improvement which directly affects patient HRQL outcomes
Scenario 6: “2-year time horizon”	Explores the consequences of different assumptions about the time horizon, as Scenario 1 with the exception that: The time horizon is set to 2 years rather than 1 year. Extrapolation of data over time has been routinely practiced in the majority of models but involves implicit assumptions. The consequences of choice of time horizon has not always been robustly tested in previous models, and results from scenario 6 will explore the consequence of simple extrapolation of assumptions over time for model outcomes

#### Sensitivity analyses

In previous models, in the absence of adequate data describing parameter uncertainty, assumptions have been necessary and have typically been based on expert opinion. However, the choice and magnitude of each assumption and its importance for results have rarely been fully justified or tested.

To characterise and assess the potential variation in model results stemming from underlying uncertainty around input parameter estimates, probabilistic sensitivity analysis (PSA) was performed using 1000 draws of input values from assumed parameter distributions. It was possible to estimate uncertainty around utility and cycle probability estimates from data reported by Ikenberg et al. [13]. For other cycle probability and cost estimates, in the absence of data, assumptions were required about the shape and scale of uncertainty around parameter values. Utility and cycle probability parameters were assumed to follow a beta distribution (bound between 0 and 1). Costs were assumed to follow a gamma distribution (non-negative and positively skewed). In previous models, particularly when expert opinion data have been used, similar assumptions have been necessary, but the choice of assumption and its importance for results have not been justified or tested. Finally, to explore the importance of assumptions about unknown parameter uncertainty, two alternative PSA runs were undertaken, assuming unknown standard errors at 50 and 10 % of the parameter estimate values, respectively.

To understand key model drivers, one-way sensitivity analyses (OWSA) were also performed. The OWSA estimated the influence of changing each uncertain parameter value between upper and lower boundaries upon the estimated incremental net benefit (INB) of novel therapy. The INB is a useful measure in sensitivity analyses when estimated outcomes can vary between positive and negative values and distort ICER (ratio) estimates, but requires a fixed willingness to pay for an additional QALY. The INB of novel therapy versus morphine is calculated here as the estimated incremental QALYs of novel therapy multiplied by a willingness to pay of £20,000 minus the estimated incremental costs of novel therapy. Again, two separate OWSA runs were undertaken, whereby one assumed unknown standard errors to be 50 % of the parameter estimate and another assumed unknown standard errors to be 10 % of the parameter estimate to demonstrate in addition the potential influence of distributional assumptions upon OWSA results in previous models.

## Results

Per-patient costs and quality-adjusted life years (QALYs) are the primary model outcomes. This exploratory analysis presents results across the two model arms described above, labelled in tables and figures as ‘morphine’ and ‘novel therapy’. Results are presented in terms of primary model outcomes and incremental outcomes across model arms.

### Scenario analyses

Table 6 shows results from the scenario analyses. The base case comparison between the novel therapy and morphine as first-line treatments for chronic pain predicts novel therapy to produce a utility gain, 0.067 more per-patient QALYs, but at a per-patient cost of over £1250. The resulting incremental cost-effectiveness ratio (ICER) for novel therapy versus morphine, which is the incremental cost over the incremental effect (QALY gain), is just over £19,125. In England and Wales, though NICE Appraisal Committees do not use a specific cost-effectiveness threshold, health technologies with a most plausible ICER of less than £20,000 per QALY gained are generally considered cost-effective, though mitigating issues including the degree of uncertainty around the ICER estimate are also considered [27]. Elsewhere in the UK and across Europe, similar approaches to health technology are prevalent, with the willingness to pay for an additional QALY varying according to jurisdiction-specific issues and budgetary constraints. This suggests a price of six times the price of oxycodone (and over 20 times the price of morphine) would be justified by a 30 % improvement in tolerability and effectiveness versus oxycodone. This ICER changes when structural and data assumptions in the model are altered, so the base case needs to be interpreted with caution.

**Table 6:** Scenario analysis results

Outcome	Morphine	Novel therapy	Incremental, novel therapy versus morphine
Scenario 1: “Base case”			
Costs	845.3130	2126.7532	£1281.44
QALYs	0.505	0.572	0.067
ICER	£19,126.66		
Scenario 2: “3rd-line treatment considered”			
Costs	£652.18	£2022.08	£1369.90
QALYs	0.536	0.589	0.053
ICER	£25,899.20		
Scenario 3: “Morphine as 2nd-line treatment on novel therapy arm”			
Costs	£845.31	£2125.49	£1280.18
QALYs	0.505	0.554	0.048
ICER	£26,550.64		
Scenario 4: “Titration and stabilisation”			
Costs	£874.29	£1867.79	£993.50
QALYs	0.490	0.561	0.070
ICER	£14,170.81		
Scenario 5: “Improvement in analgesic effect”			
Costs	£845.31	£2126.75	£1281.44
QALYs	0.505	0.591	0.085
ICER	£15,000.22		
Scenario 6: “2-year time horizon”			
Costs	£1787.01	£3390.83	£1603.82
QALYs	0.864	0.996	0.132
ICER	£12,182.50		
Scenario 7: “No assumed HRQL decrement over successive treatment lines”			
Costs	£845.31	£2126.75	£1281.44
QALYs	0.557	0.603	0.046
ICER	£27,970.41		

When assumptions about the care pathway beyond the first line are altered (scenarios 2 and 3) the ICER estimate varies substantially. The model state “Subsequent treatment” is associated with a lower cycle cost and higher utility tariff than “Treatment discontinuation”, and accordingly per-patient costs are lower while QALYs are higher across both model arms in scenario 2 compared to scenario 1. Despite this adjustment being applied to both model arms, due to higher withdrawal rates in the morphine arm, the ICER increases to nearly £25,900 in scenario 2.

In scenario 3, patients in the novel therapy arm are assumed to receive morphine, not oxycodone, as a second-line therapy. The input data used imply a lower cost but higher withdrawal rates for morphine versus oxycodone, the base case second-line treatment, and total costs and QALYs on the novel therapy arm are reduced accordingly in scenario 3. As a result, the estimated ICER increases to over £26,500. When there is uncertainty about the treatment pathway, it is clearly important to explore how such uncertainty may affect results.

In scenario 4, costs and treatment costs and cycle probabilities of withdrawal were adjusted for the first four model cycles to capture the lower treatment dose and increased withdrawal rates observed in clinical practice during the titration period. Per-patient QALYs are reduced across both arms relative to scenario 1, reflecting higher withdrawal rates in the first 4 weeks of treatment. Per-patient costs are lower than the base case for the novel therapy arm and reduced to a lesser extent in the morphine arm. This is explained by the higher treatment costs of novel therapy versus oxycodone and oxycodone versus morphine and the relative treatment cost savings from a proportional reduction in dose across model arms during the titration and stabilisation phase. The ICER is reduced by nearly £5000 compared to scenario 1.

In scenario 5, the 5 % improvement in utility attributed to first-line treatment with novel therapy to reflect a potential improvement in analgesia leads to a 50 % increase in the incremental QALYs associated with the novel therapy arm compared to the base case in scenario 1. As a result, the ICER estimate is driven down by over £4000. Forthcoming therapeutic agents may offer analgesic benefit, and this could clearly have a substantial impact upon cost-effectiveness estimates.

In scenario 6, the model time horizon was increased to 2 years. This caused the ICER estimate to fall to just under £12,200. After 1 year, the base case model estimates that over 50 % of patients will still be receiving either first- or second-line therapy in the novel therapy arm, whereas this figure is <30 % in the morphine arm. The relative benefit of receiving novel therapy versus morphine as a first-line treatment resonates beyond 1 year; this highlights how assumptions used to extrapolate data in economic models can be of importance for study results.

In scenario 7, ignoring potential lasting reductions in patient HRQL associated with experiencing treatment failure leads to a 30 % reduction in the expected incremental QALY gain associated with the novel therapy, compared to the base case. As a result, the ICER estimate is increased to nearly £28,000. Model results are clearly highly sensitive to assumptions about patient HRQL, an area in which data are lacking.

### Sensitivity analyses

**Fig. 2:**
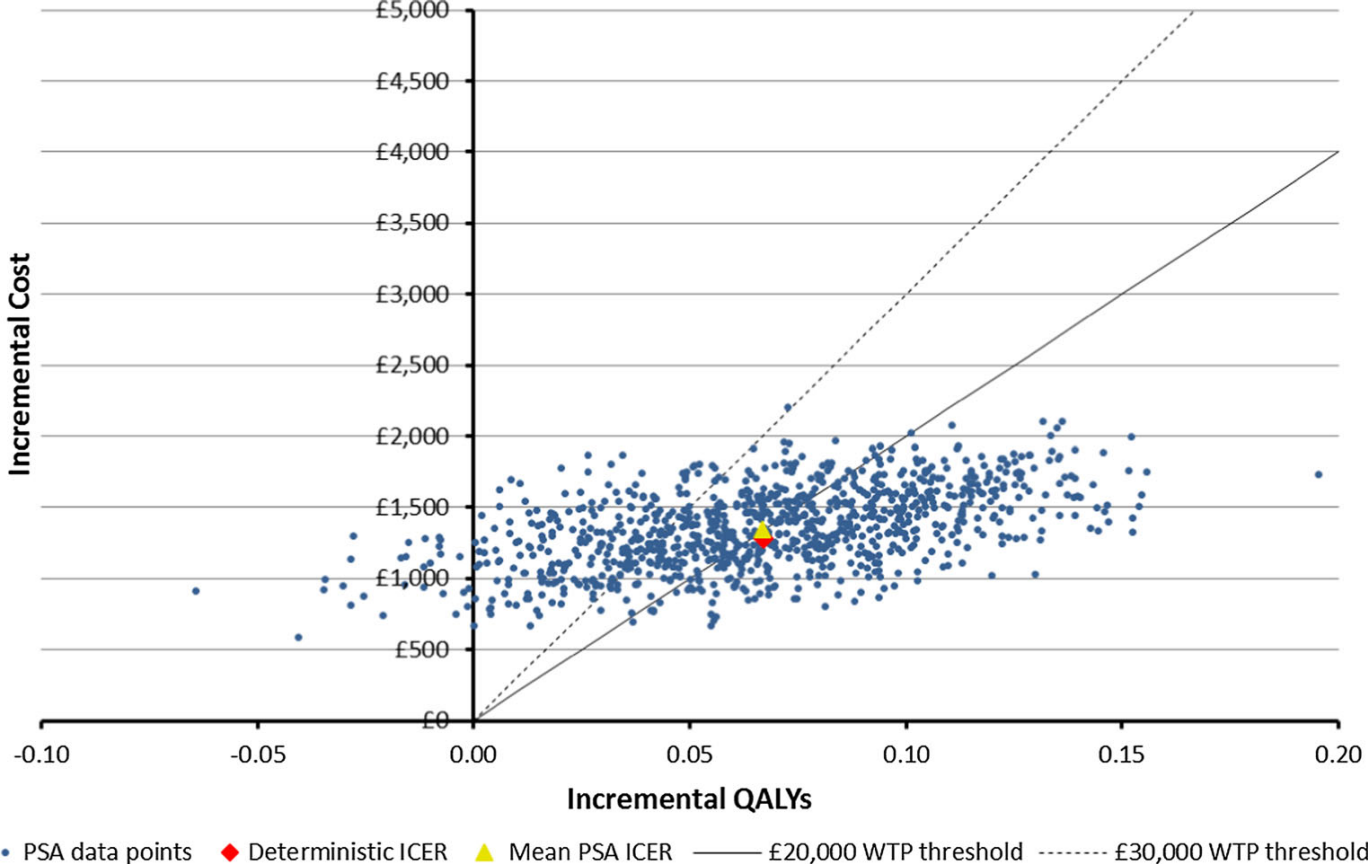
Scatterplot of base case cost-effectiveness pairs, assuming 50 % standard errors

**Fig. 3:**
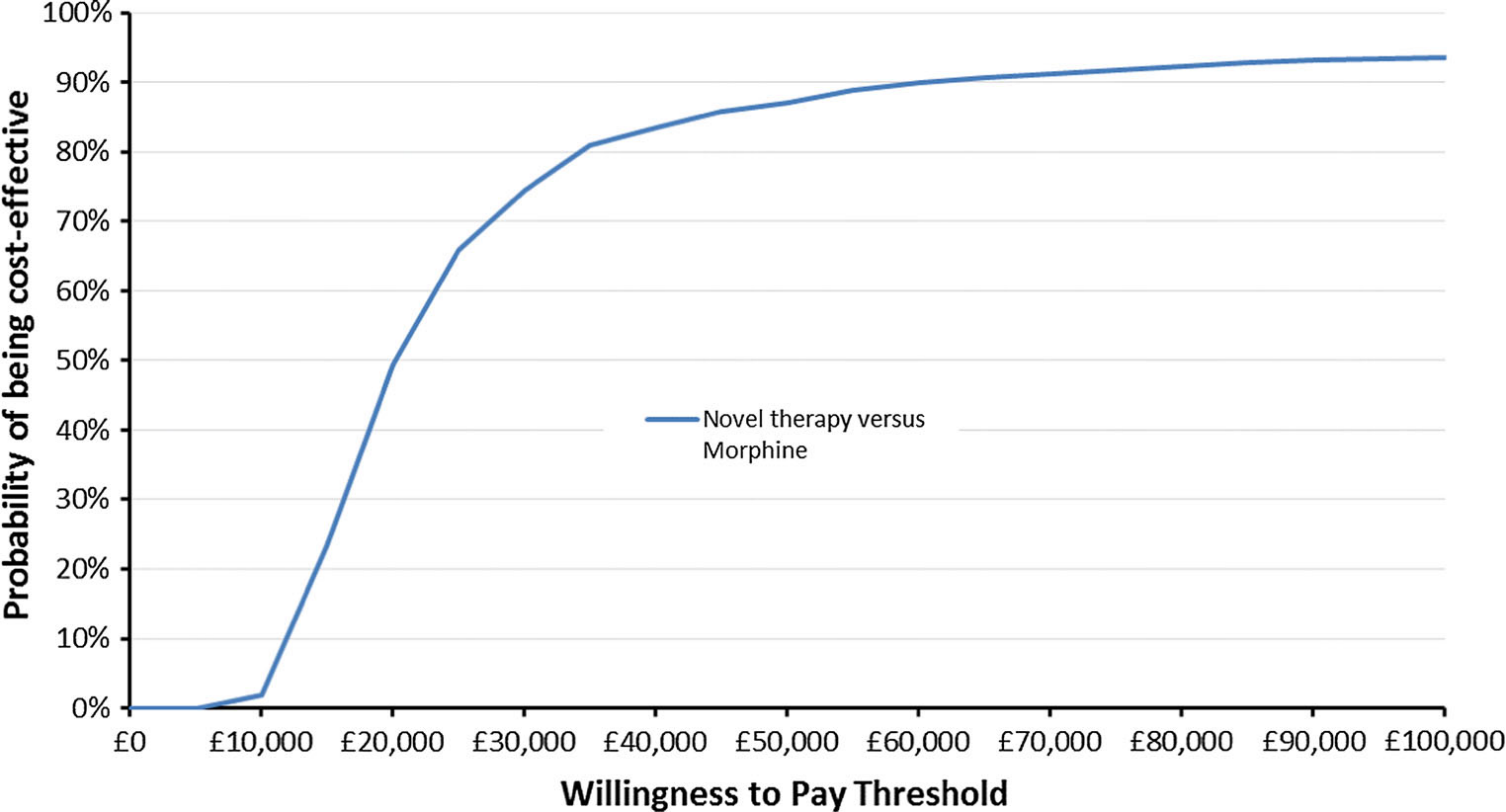
Cost-effectiveness acceptability curve using the Fig. 2 results

**Fig. 4:**
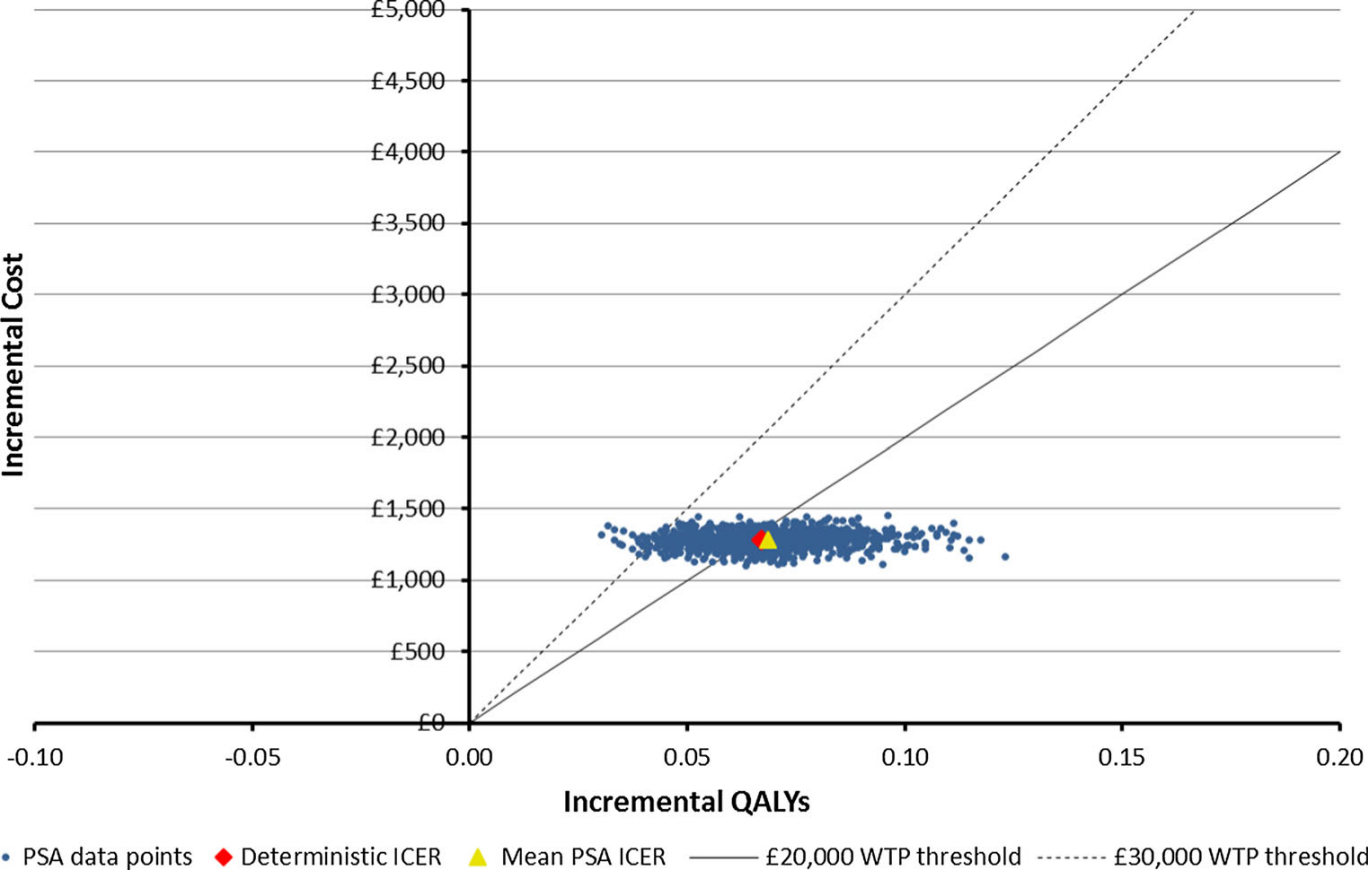
Scatterplot of base case cost-effectiveness pairs, base case, assuming 10 % standard errors

**Fig. 5:**
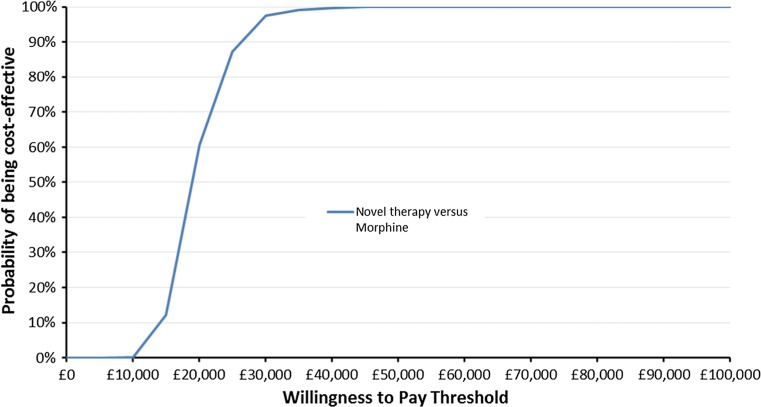
Cost-effectiveness acceptability curve using the Fig. 4 results

Figure 2 shows scatterplots of 1000 PSA ICER estimates where the standard error of parameter point estimate values with unknown distributions is assumed to be 50 % of the parameter point estimate. Figure 4 shows the corresponding PSA ICER estimate scatterplot where these standard errors are assumed to be 10 % of the parameter point estimate. Figures 3 and 5 show CEACs produced using the data presented in Figs. 2 and 4, respectively.

Comparing Figs. 2 and 3 with Figs. 4 and 5, it is clear that assumptions about the scale of uncertainty around parameter estimates can influence the apparent uncertainty around model outcomes. Viewing Figs. 2 and 3, the incremental QALY gain associated with novel therapy ranges from −0.07 to over 0.19 across PSA draws and at a willingness to pay of £30,000 for an additional QALY, the probability novel therapy is preferable to morphine as a first-line treatment is shown to be 74 %. By contrast, Figs. 4 and 5 show no PSA draw at which the morphine arm produced the greater estimated health outcome and suggest a 98 % probability that novel therapy is preferable to morphine as a first-line treatment, at a willingness to pay of £30,000 for an additional QALY. Where previous models have suggested their results are robust to sensitivity tests this may have been primarily due to underestimates of uncertainty around highly uncertain parameters.

**Fig. 6:**
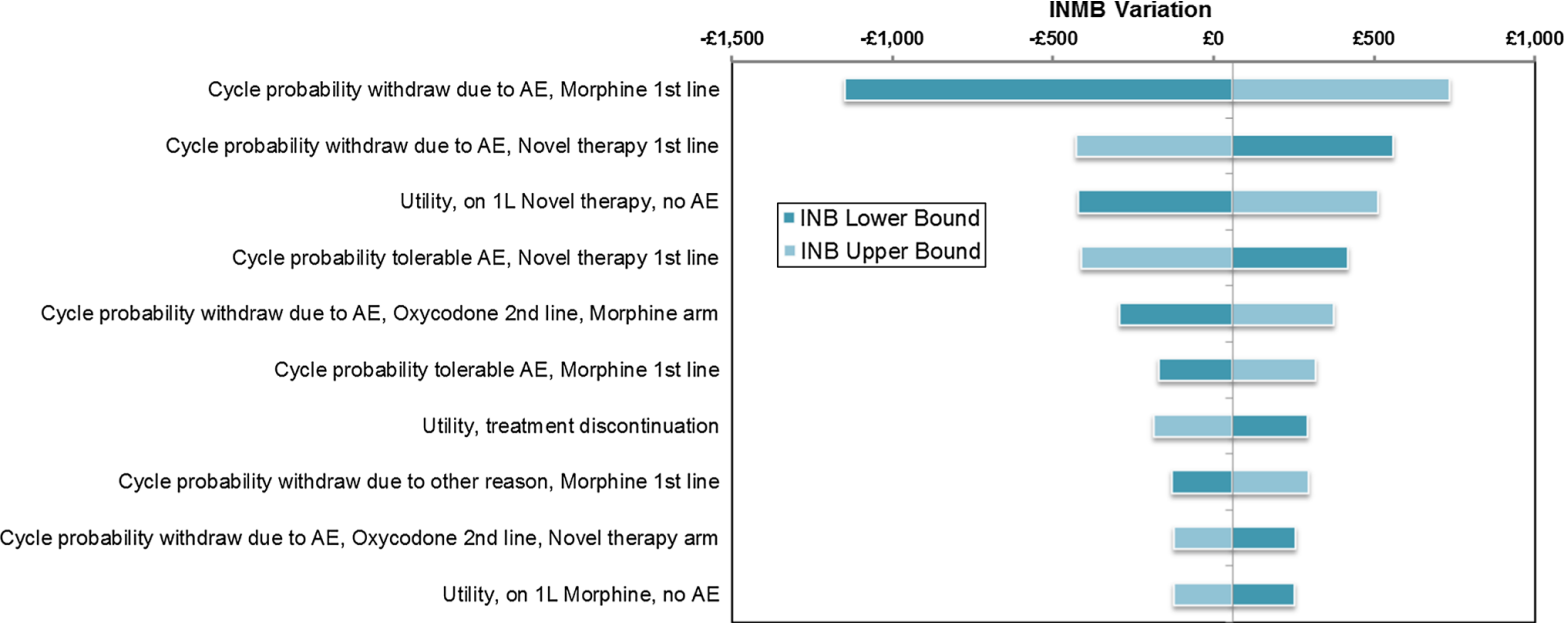
Tornado diagram of the top ten most influential parameters from OWSA, assuming 50 % standard errors

**Fig. 7:**
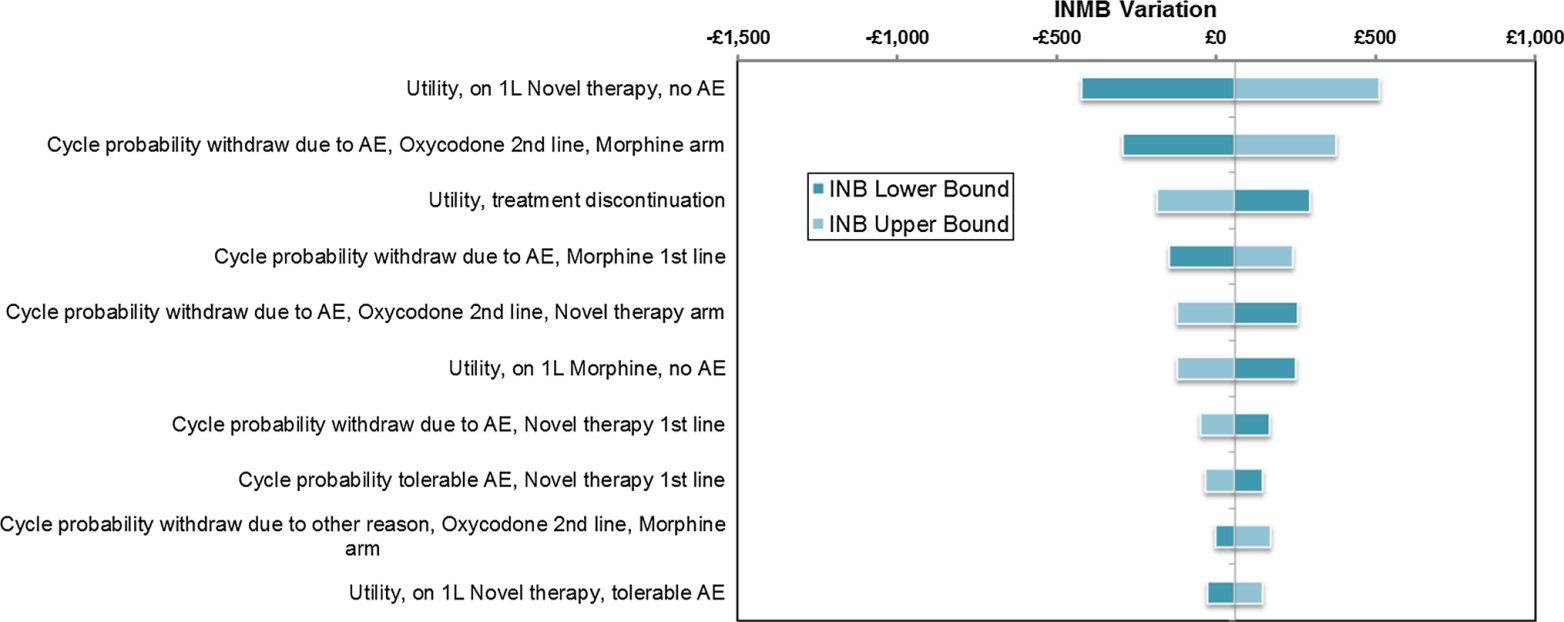
Tornado diagram of the top ten most influential parameters from OWSA, assuming 10 % standard errors

Figures 6 and 7 show tornado diagrams presenting results from the OSWA run under different assumptions about the scale of uncertainty around parameter estimates. The model is sensitive to uncertainty around the HRQL of patients on first-line treatment who are not experiencing tolerable AEs, which is wide as only 215 patients in the sample of Ikenberg et al. [13] informed this estimate. The relative and absolute importance of uncertainty around cycle probabilities of treatment withdrawal is clearly determined by assumptions about the scale of parameter uncertainty in Figs. 6 and 7. Where previous models have suggested that their results are insensitive to changes in highly uncertain parameter inputs, this may have been primarily due to arbitrary assumptions about the scale of uncertainty around parameter estimates.

## Discussion

The need across Europe to justify resource allocation decisions based on economic value has placed a burden on manufacturers to provide evidence of value using robust modelling approaches and data. In an area such as chronic pain management, key robust data are scarce, so this task is more difficult. In order to increase the chance of correct societal resource allocation decisions and maintain incentives for continued development of analgesic technologies, it is vital that modelling approaches are both transparent and robust.

The systematic literature review identified key areas of modelling variability and reliance on assumptions in previous chronic pain models. Past models have varied in their structural complexity; more complex models have generally been reliant on expert data to inform model parameters to a greater extent. The scenarios explored in this study using the de novo reference model highlight how certain structural and data assumptions may have influenced cost-effectiveness results in previous economic evaluations of treatments for chronic pain. Economic evaluations are a necessary medium to justify price premiums for novel therapies, but European decision bodies such as NICE may have been distrustful of previous model results because of the absence of robust exploration of the implications of uncertainty around these assumptions. Though non-specific in their criticism in a review of evidence for opioids in palliative care, a NICE GDG described three studies identified here [6, 11, 12] as subject to potentially serious limitations [10]. Each of these studies required assumptions and expert opinion to structure and parameterise their model; if the uncertainty around these assumptions had been robustly explored, the NICE GDG may have found evidence from previous studies of far more use.

Different structural and parameter assumptions explored in this study were shown to influence cost-effectiveness estimates, with the deterministic ICER estimate ranging from around £12,000 when a time horizon of 2 years was used to over £25,000 when different assumptions about the care pathway were explored. There is variation in treatment patterns for chronic pain, and it is clearly vitally important that the consequences of this variation and uncertainty are robustly tested in future economic models.

The economic evaluations of chronic pain therapies identified in the systematic literature review considered opioid therapies only. Perhaps due to the trial design in opioid studies, whereby dosing is carefully adjusted to balance the analgesic effect with tolerability, in previous models the incremental value between treatments has been judged on utility gain from differences in the tolerable and intolerable adverse event rate rather than differences in the analgesic effect. Common practice has instead been to account for differences in analgesia more crudely by accounting for withdrawal due to efficacy. Future therapies may also offer improvements in analgesia, regardless of dose. This study has explored the consequences of a relative improvement in analgesic effect for one treatment versus another for cost-effectiveness estimates, and the results suggest that cost-effectiveness estimates are sensitive to relative utility scores across comparator arms. However, it may be important in future studies to robustly quantify with greater sensitivity the relationship between pain outcome measures, such as the widely used 11-point numerical rating scale (NRS), and patient HRQL. Two previous studies have investigated the relationship between the EQ-5D and the 11-point NRS in general population *(n* = 100) [28] and neuropathic pain patient (*n* = 284) [29] samples.

The analysis presented here is subject to limitations. The input data for the reference model were sourced from the systematic review of previous economic analyses, and these were likely not ultimately the best available input data for a definitive HTA model in pain. Nevertheless, the plausibility of inputs has been validated by a clinical expert. Future research could involve greater exploration of the treatment pathway—particularly differences between geographies. Further, several previous studies have identified input data through systematic searches [6, 10, 12, 13, 18] yet were still reliant on expert opinion data to varying degrees. Data availability from previous models may be a strong indicator of data availability in the field. In addition, though this study has sought to identify and test assumptions around key decision points in the modelling of chronic pain, the importance of different methodological approaches that vary across jurisdictions, such as consideration of indirect costs, has not been tested. Calculating indirect costs related to pain is a very important area of future research, particularly given many patients suffering from pain are of working age and the high prevalence of chronic pain in the general population.

Economic evaluation to inform treatment choices in chronic pain is important but challenging, not least because of data limitations. This study has highlighted the pressing need for better knowledge on outcomes for patients after they withdraw from treatment. Trial designs to capture patient HRQL and other outcomes over multiple treatment lines would be of great value. If expert opinion is required, it is also important that the method of elicitation is robust; guidance and recommendations for expert elicitation in health economics are available [30]. Finally, further versions of chronic pain models could account for other important differentiators of pain therapy—addiction/abuse potential and respiratory depression being just two examples.

## Conclusion

Due to data limitations characteristic to chronic pain, it is critically important that future models in chronic pain are designed to be fully transparent in order to aspire to a common approach to modelling pain and must include a robust and well-designed set of reported sensitivity analyses. This is important for fair and consistent decision making from both the patient perspective and also those needing to invest in new therapies. We hope that the open-source reference model structure, as reported in this article, can act as the initial step in the development of a more consistent and transparent reference point for the development and assessment of future economic models in pain.

## Electronic supplementary material

Below is the link to the electronic supplementary material.

Supplementary material 1 (DOCX 33 kb)

Supplementary material 2 (XLSM 1312 kb)

Supplementary material 3 (R 104 kb)

Supplementary material 4 (R 25 kb)

## Footnotes

1 The different treatment pathway for chronic neuropathic pain compared to other chronic pain means that it falls outside of the scope of this study.
