# Supplementary material for: Economic evaluation in chronic pain: a systematic review and de novo flexible economic model
Source: Eur J Health Econ. 2015 Sep 16;17:755–70. doi: 10.1007/s10198-015-0720-y (PMC4899502; doi:10.1007/s10198-015-0720-y)
Supplement: Supplementary file 1 — Supplementary material 1 (DOCX 33 kb) [file 10198_2015_720_MOESM1_ESM.docx]

Appendix: Literature Review Search Strategy and Additional Results

*Search Strategy and Database Results*

**Searches Conducted 22/04/2014-23/04/2014**

| **Database** | **Cost-effectiveness ($$cost-effectiveness)** |
| --- | --- |
| MEDLINE and MEDLINE In-Process via Ovid 1946-Present ($$medline) | 171 |
| EMBASE via Ovid 1974-Present ($$embase) | 617 |
| HTA via The Cochrane Library 1989-Present ($$hta) | 11 |
| NHS EED via The Cochrane Library 1968-Present | 59 |
| EconLit | 2 |
| Total references retrieved | 860 |
| **Total unique** **references** | **689** |

*PRISMA diagram*


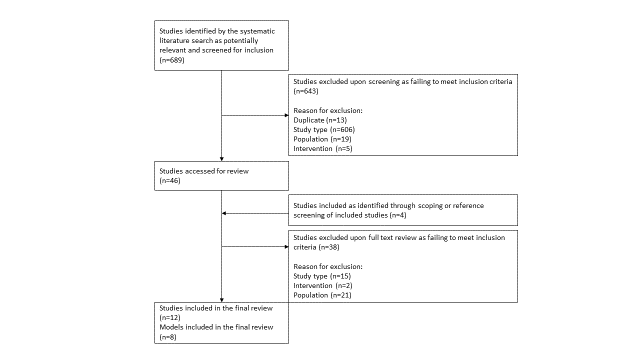


*Search Strategies*

**MEDLINE**

***Population Terms (1-24)***

1. *Neuralgia/

2. nonmalignant pain.ti,ab.

3. severe pain.ti,ab.

4. neuropath*.ti,ab.

5. neuralgia*.ti,ab.

6. neurodynia*.ti,ab.

7. nerve pain*.ti,ab.

8. *Chronic Pain/

9. chronic pain*.ti,ab.

10. *Pain/

11. pain*.ti.

12. 10 or 11

13. *Neoplasms/

14. neoplas*.ti,ab.

15. tumo?r*.ti,ab.

16. cancer*.ti,ab.

17. elderly.ti,ab.

18. *Aged/

19. *"Aged, 80 and over"/

20. *Frail Elderly/

21. 13 or 14 or 15 or 16 or 17 or 18 or 19 or 20

22. 12 and 21

23. or/1-9

24. 22 or 23

***Intervention Terms (25-44)***

25. pregabalin.mp.

26. gabapentin.mp.

27. tapentadol.mp.

28. fentadyl.mp.

29. oxycodone.mp. or Oxycodone/

30. morphine.mp. or Morphine/

31. amitriptyline.mp. or Amitriptyline/

32. duloxetine.mp.

33. tramadol.mp. or Tramadol/

34. carbamazepine.mp. or Carbamazepine/

35. capsaicin.mp. or Capsaicin/

36. buprenorphine.mp. or Buprenorphine/

37. diamorphine.mp.

38. hydromorphone.mp. or Hydromorphone/

39. hydrocodone.mp. or Hydrocodone/

40. Oxymorphone/ or oxymorphone.mp.

41. codeine.mp. or Codeine/

42. dihydrocodeine.mp.

43. paracetamol.mp. or Acetaminophen/

44. aspirin.mp. or Aspirin/

45. or/25-44

46. 24 and 45

***SIGN Economics Studies Filter (47-78)***

47. Economics/

48. "costs and cost analysis"/

49. Cost allocation/

50. Cost-benefit analysis/

51. Cost control/

52. cost savings/

53. Cost of illness/

54. Cost sharing/

55. "deductibles and coinsurance"/

56. Health care costs/

57. Direct service costs/

58. Drug costs/

59. Employer health costs/

60. Hospital costs/

61. Health expenditures/

62. Capital expenditures/

63. Value of life/

64. exp economics, hospital/

65. exp economics, medical/

66. Economics, nursing/

67. Economics, pharmaceutical/

68. exp "fees and charges"/

69. exp budgets/

70. (low adj cost).mp.

71. (high adj cost).mp.

72. (health?care adj cost$).mp.

73. (fiscal or funding or financial or finance).tw.

74. (cost adj estimate$).mp.

75. (cost adj variable).mp.

76. (unit adj cost$).mp.

77. (economic$ or pharmacoeconomic$ or price$ or pricing).tw.

78. or/47-77

79. 46 and 78

80. limit 79 to (english language and humans and yr="2000 -Current")

**EMBASE**

***Population Terms (1-25)***

1. *neuralgia/

2. nonmalignant pain.ti,ab.

3. severe pain.ti,ab.

4. neuropath*.ti,ab.

5. neuralgia*.ti,ab.

6. neurodynia*.ti,ab.

7. nerve pain*.ti,ab.

8. *chronic pain/

9. chronic pain*.ti,ab.

10. *pain/

11. pain*.ti.

12. 10 or 11

13. *neoplasm/

14. neoplas*.ti,ab.

15. tumo?r*.ti,ab.

16. cancer*.ti,ab.

17. elderly.ti,ab.

18. *Aged/

19. *"Aged, 80 and over"/

20. *Frail Elderly/

21. *very elderly/

22. 13 or 14 or 15 or 16 or 17 or 18 or 19 or 20 or 21

23. 12 and 22

24. or/1-9

25. 23 or 24

***Intervention Terms (26-46)***

26. pregabalin.ti,ab. or *pregabalin/

27. gabapentin.ti,ab. or *gabapentin/

28. tapentadol.ti,ab. or *tapentadol/

29. fentadyl.mp.

30. oxycodone.ti,ab. or *oxycodone/

31. *morphine/ or morphine.ti,ab.

32. *amitriptyline/ or amitriptyline.ti,ab.

33. duloxetine.ti,ab. or *duloxetine/

34. *tramadol/ or tramadol.ti,ab.

35. *carbamazepine/ or carbamazepine.ti,ab.

36. capsaicin.ti,ab. or *capsaicin/

37. buprenorphine.ti,ab. or *buprenorphine/

38. diamorphine.ti,ab. or *diamorphine/

39. hydromorphone.ti,ab. or *hydromorphone/

40. *hydrocodone/ or hydrocodone.ti,ab.

41. oxymorphone.ti,ab. or *oxymorphone/

42. *codeine/ or codeine.ti,ab.

43. *dihydrocodeine/ or dihydrocodeine.ti,ab.

44. *paracetamol/ or paracetamol.ti,ab.

45. aspirin.ti,ab. or *acetylsalicylic acid/

46. or/26-45

47. 25 and 46

***SIGN Economics Studies Filter (48-65)***

48. exp SOCIOECONOMICS/

49. exp "Cost Benefit Analysis"/

50. exp "Cost Effectiveness Analysis"/

51. exp "Cost of Illness"/

52. exp "Cost Control"/

53. exp Economic Aspect/

54. exp Financial Management/

55. exp "Health Care Cost"/

56. exp Health Care Financing/

57. exp Health Economics/

58. exp "Hospital Cost"/

59. (financial or fiscal or finance or funding).tw.

60. exp "Cost Minimization Analysis"/

61. (cost adj estimate$).mp.

62. (cost adj variable$).mp.

63. (unit adj cost$).mp.

64. or/48-63

65. 47 and 64

66. limit 65 to (human and english language and yr="2000 -Current")

**The Cochrane Library (HTA, NHS EED)**

***Population Terms (1-24)***

1 MeSH descriptor: [Neuralgia] this term only

2 nonmalignant pain:ti or nonmalignant pain:ab

3 severe pain:ti or severe pain:ab

4 neuropath*:ti or neuropath*:ab

5 neuralgia*:ti or neuralgia*:ab

6 neurodynia*:ti or neurodynia*:ab

7 nerve pain*:ti or nerve pain*:ab

8 MeSH descriptor: [Chronic Pain] this term only

9 chronic pain*:ti or chronic pain*:ab

10 MeSH descriptor: [Pain] this term only

11 pain*:ti or pain*:ab

12 10 or 11

13 MeSH descriptor: [Neoplasms] this term only

14 neoplas*:ti or neoplas*:ab

15 tumo?r*:ti or tumo?r*:ab

16 cancer*:ti or cancer*:ab

17 elderly:ti or elderly:ab

18 MeSH descriptor: [Aged] this term only

19 MeSH descriptor: [Aged, 80 and over] this term only

20 MeSH descriptor: [Frail Elderly] this term only

21 or 13-20

22 12 and 21

23 or 1-9

24 22 or 23

***Intervention Terms (25-59)***

25 pregabalin

26 gabapentin

27 tapentadol

28 fentadyl

29 MeSH descriptor: [Oxycodone] explode all trees

30 oxycodone

31 MeSH descriptor: [Morphine] explode all trees

32 morphine

33 amitriptyline

34 MeSH descriptor: [Amitriptyline] explode all trees

35 Amitriptyline

36 duloxetine

37 tramadol

38 MeSH descriptor: [Tramadol] explode all trees

39 carbamazepine

40 MeSH descriptor: [Carbamazepine] explode all trees

41 capsaicin

42 MeSH descriptor: [Capsaicin] explode all trees

43 buprenorphine

44 MeSH descriptor: [Buprenorphine] explode all trees

45 diamorphine

46 MeSH descriptor: [Hydromorphone] explode all trees

47 hydromorphone

48 MeSH descriptor: [Hydrocodone] explode all trees

49 hydrocodone

50 MeSH descriptor: [Oxymorphone] explode all trees

51 oxymorphone

52 codeine

53 MeSH descriptor: [Codeine] explode all trees

54 dihydrocodeine

55 paracetamol

56 MeSH descriptor: [Acetaminophen] explode all trees

57 aspirin

58 MeSH descriptor: [Aspirin] explode all trees

59 or 25-58

60 24 and 59

Publication Date from 2000 to 2014

**EconLit**

***Population Terms (1-16)***

1. nonmalignant pain.ti,ab.

2. severe pain.ti,ab.

3. neuropath*.ti,ab.

4. neuralgia*.ti,ab.

5. neurodynia*.ti,ab.

6. nerve pain*.ti,ab.

7. chronic pain*.ti,ab.

8. or/1-7

9. pain*.ti.

10. neoplas*.ti,ab.

11. tumo?r*.ti,ab.

12. cancer*.ti,ab.

13. elderly.ti,ab.

14. or/10-13

15. 9 and 14

16. 8 or 15

***Intervention Terms (17-37)***

17. pregabalin.mp.

18. gabapentin.mp.

19. tapentadol.mp.

20. fentadyl.mp.

21. oxycodone.mp.

22. morphine.mp.

23. amitriptyline.mp.

24. duloxetine.mp.

25. tramadol.mp.

26. carbamazepine.mp.

27. capsaicin.mp.

28. buprenorphine.mp.

29. diamorphine.mp.

30. hydromorphone.mp.

31. hydrocodone.mp.

32. oxymorphone.mp.

33. codeine.mp.

34. dihydrocodeine.mp.

35. paracetamol.mp.

36. aspirin.mp.

37. or/13-32

38. 16 and 37

39. limit 38 to (english language and yr="2000 -Current")

***Search Filters Used***

SIGN Economics Studies Filters: [http://www.sign.ac.uk/methodology/filters.htmlecon](http://www.sign.ac.uk/methodology/filters.html#econ)
